# Specific absorption rate and temperature in neonate models resulting from exposure to a 7T head coil

**DOI:** 10.1002/mrm.28784

**Published:** 2021-04-03

**Authors:** Shaihan J. Malik, Jeffrey W. Hand, Ryan Satnarine, Anthony N. Price, Joseph V. Hajnal

**Affiliations:** 1 Department of Biomedical Engineering, School of Biomedical Engineering and Imaging Sciences, King’s College London, St. Thomas’ Hospital, London, United Kingdom; 2 Center for the Developing Brain, School of Biomedical Engineering and Imaging Sciences, King’s College London, St. Thomas’ Hospital, London, United Kingdom

**Keywords:** 7T MRI, neonatal imaging, RF safety

## Abstract

**Purpose:**

To investigate safe limits for neonatal imaging using a 7T head coil, including both specific absorption rate (SAR) and temperature predictions.

**Methods:**

Head-centered neonate models were simulated using finite-difference time domain–based electromagnetic and thermal solvers. The effects of higher water content of neonatal tissues compared with adults, position shifts, and thermal insulation were also considered. An adult model was simulated for comparison.

**Results:**

Maximum and average SAR are both elevated in the neonate when compared with an adult model. When normalized to B_1_^+^, the SAR experienced by a neonate is greater than an adult by approximately a factor of 2; when normalized to net forward power (forward-reflected), this increases to a factor of 2.5-3.0; and when normalized to absorbed power, approximately a factor of 4. Use of age-adjusted dielectric properties significantly increases the predicted SAR, compared with using adult tissue properties for the neonates. Thermal simulations predict that change in core temperature/maximum temperature remain compliant with International Electrotechnical Commission limits when a thermally insulated neonate is exposed at the SAR limit for up to an hour.

**Conclusion:**

This study of two neonate models cannot quantify the variability expected within a larger population. Likewise, the use of age-adjusted dielectric properties have a significant effect, but while their use is well motivated by literature, there is uncertainty in the true dielectric properties of neonatal tissue. Nevertheless, the main finding is that unlike at lower field strengths, operational limits for 7T neonatal MRI using an adult head coil should be more conservative than limits for use on adults.

## INTRODUCTION

The use of MRI in studying normal and abnormal brain development in neonates is of great interest. Such imaging demands sufficient spatial resolution to resolve small brain structures within acceptable acquisition times, while maintaining safe exposures.^1^ Use of ultrahigh-field MRI, for example at 7 T, has the potential to deliver the enhanced sensitivity and resolution needed to explore neonatal brain development and pathology. However, the increase of specific absorption rate (SAR) with frequency and the exclusive use of local transmit coils means that safe operation cannot be assumed; currently, there are no European Conformity ('CE marked') or Food and Drug Administration–approved methods for imaging of neonates at 7 T. Although there are numerous reports of imaging adults using ultrahigh-field MRI, reports of imaging of young children and neonates in systems >3 T are sparse.^2,3^ The Food and Drug Administration currently advises that static fields above 4 T pose a significant risk to subjects younger than 1 month^4^; the International Electrotechnical Commission (IEC)^5^ guidelines do not specifically mention neonates, but classify 4 T to 8 T as first-level operating mode. Nevertheless, the application of 7T MRI to neonates has recently been reported.^6^

The hazard from RF energy deposition comes from resulting temperature increases that, along with SAR, are subject to limits defined by an international standard.^5^ In addition to their small size, neonates differ from adults in that their tissues have a higher water content, which is dependent on age, and this is known to affect their dielectric properties. Reliable measurements of human neonatal tissue dielectric properties are not widely available, and instead, many existing studies have simply assumed that neonatal tissues have the same dielectric properties as adults.^6^ In a previous study at lower field strengths, we found that neonates are less prone to RF heating when using large birdcage transmitters.^7^

Accurate predictions of resulting temperature increases in neonates are hampered due to their underdeveloped thermal regulation and uncertainty in the thermal properties of neonatal tissues. Unlike adults, who are often modeled as being thermally stable when unclothed at room temperature, heat loss associated with neonates’ high surface-to-volume ratios causes them to become hypothermic in this scenario.^8^

In this safety study for neonatal ultrahigh-field MRI, simulations involving a generic 7T (adult) head transmit coil and two neonate voxel models have been investigated. One of the voxel models was created as part of this study, based on MRI data of a neonate with a posture that is typical of that adopted by such subjects. Both adult and neonatal tissue dielectric properties were used in performing RF simulations, with the latter determined by scaling adult properties to adjust for differences in water content using methods taken from literature. In an initial attempt to represent a thermally stable neonate in a background temperature of 22°C, representative of the MRI environment, thermal simulations of the novel voxel model included a “blanket” layer used to simulate thermal insulation, as is often used in practice.

## 2 METHODS

### 2.1 Voxel models

**Figure 1:**
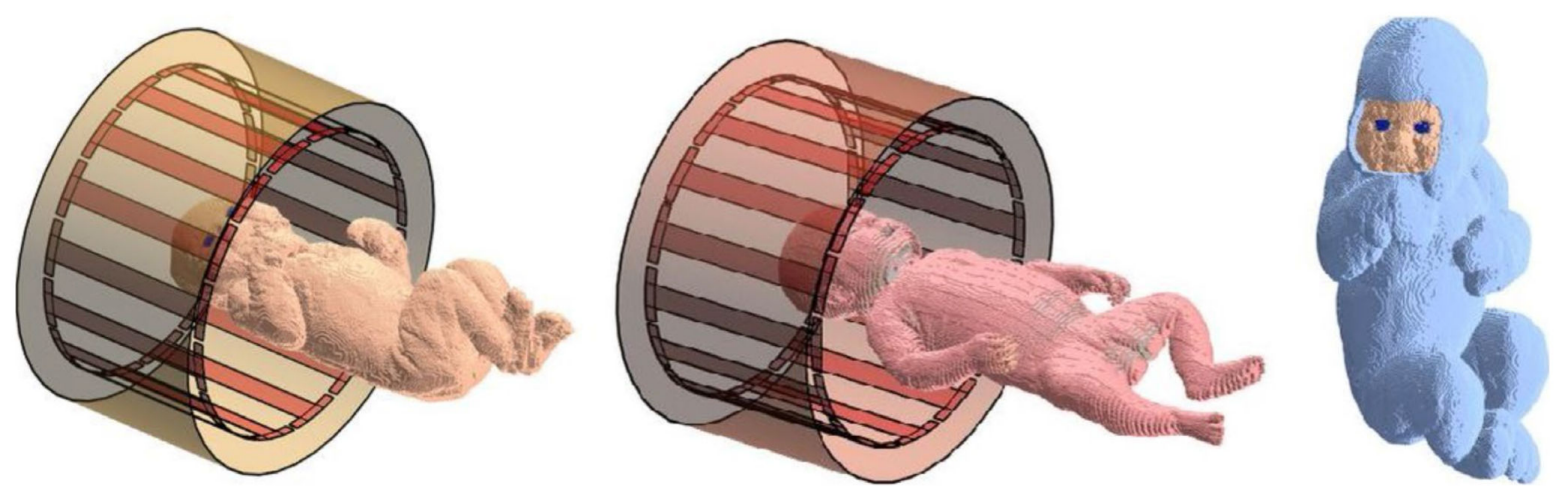
Neonates within transmit coil and shield. Left: Neonate A. Center: Neonate B. The large shield representing the bore of the scanner is not shown. Right: Neonate A with insulating “blanket”

Two neonate models were included in this work. Neonate A was a new model created from Dixon 3T MRI images^9,10^ of a neonate (female; gestational age, 38 weeks 6 days; age at scan, 1 day; birth weight, 3.50 kg) acquired at St. Thomas’ Hospital, London, following written consent according to local ethics arrangements. Using these images and the open-source image analysis tool ITK-SNAP,^11^ a segmented model with 13 tissue types and resolution of 0.98 × 0.98 × 1.5 mm^3^ was created. Postprocessing, which included segmentation of the skin and smoothing of the segmented tissues, was implemented using *MATLAB* (The MathWorks, Natick, MA). The second neonate model (neonate B) was a modified version of an 8-week-old model based on postmortem CT data, segmented into 31 tissue types, obtained from the Helmholtz Zentrum München—German Research Center for Environmental Health.^12,13^ Following previous work,^7^ the original data were modified by scaling the voxel size down by 4% in both transverse directions and 14% in the superior– inferior direction, to be more representative of a term infant. Figure 1 shows neonates A and B positioned brain-centered within the birdcage coil. The two orthogonal ports are located within the end-ring proximal to the neonate’s head. Details of the weight (calculated using database values of tissue density) and length of the two neonate models and statistics relative to the World Health Organization child growth standards^14^ are given in Supporting Information Table S1. Finally, a simulation of an adult male (Duke v3.1; Zurich MedTech, Zurich, Switzerland) brain-centered within the coil was performed for comparison with the neonate simulations. Head regions used for computing head-averaged SAR were manually defined: volumes in neonates A and B and the adult model were 1030 mL, 800 mL, and 4800 mL, respectively.

### 2.2 Transmit coil model

The 7T high-pass birdcage head coil was 190 mm long, 305 mm in diameter, with 16 rungs. It was surrounded by a shield that was 205 mm long and 370 mm in diameter. The coil was driven in quadrature using two orthogonal ports (with fixed 90° phase difference) in one end ring and tuned by inserting capacitors across gaps in both end rings located between the rungs. To reduce the power radiated from the coil and shield, these structures were placed centrally within a larger cylindrical shell 1500 mm long and 650 mm in diameter, representing the bore of the MR scanner. All conductors were assumed to be copper with a conductivity of 5.997 × 10^7^ S m^−1^.

### 2.3 Numerical simulations

Electromagnetic (EM) and thermal simulations were carried out using *Sim4Life* v5.0.0 (Zurich MedTech) on PCs with 3.6-GHz Intel Core i7-3820 processors and 16 GB of RAM. Acceleware finite-difference time-domain solvers (Calgary, Canada) on Tesla C2075 GPU cards with 6 GB of memory (Nvidia, Santa Clara, CA) were used for EM simulations. Uniaxial perfectly matched layers, absorbing boundary conditions set to “medium,” were used at the edges of the computation domain. Autogridding was used, which resulted in minimum and maximum steps and voxel counts for the neonate and Duke simulations, as indicated in Supporting Information Table S3. Simulation times were set to 100 periods, and autotermination was set to strict (−75 dB).

Wideband excitation was investigated using a Gaussian pulse with center frequency of 297 MHz and bandwidth of 100 MHz when Duke was positioned with the corpus collo-sum at the center of the coil. Harmonic simulations at 297 MHz with the same coil tuning were then carried out for the neonate cases (ie, the coil was not retuned from the adult, as would be the case in practice).

### 2.4 Dielectric properties

An age dependence of neonatal dielectric properties is to be expected, given the age dependence of tissue–water content,^15–17^ although benchmark human neonatal values are not widely available. The dielectric properties required for the neonatal models used in this study were derived using a similar approach to that reported by Dimbylow et al,^18^ in which adult dielectric properties were scaled by ratios calculated between the dielectric properties of newborn and adult rats.^15^ Supporting Information Table S2 lists the ratios at 297 MHz and the resulting dielectric properties for newborn tissues used in the two models, with adult values taken from Hasgall et al.^19^

Simulations were also run using adult tissue properties for comparison. In light of the greater degree of tissue segmentation in neonate B compared with that of neonate A, simulation of neonate B (with adult properties) was repeated with tissues not segmented in neonate A, with all tissues not segmented in neonate A allocated properties of connective tissue.

### 2.5 Thermal simulations

Thermal simulations were carried out for neonate A (with age-adjusted dielectric properties) and the adult model. Unlike adults, neonates are not in thermal equilibrium with their surroundings, if naked at room temperature. The small blood pool volume (~300 mL) means that core temperature changes are likely to occur, whereas such an effect in adults is usually very small, particularly when local transmit conditions apply.

Thermal simulations were implemented by solving the bioheat equation^20^ using the finite-difference time-domain solver within the *Sim4Life* software package, including use of variable core temperature in some cases. Technical details of how this has been implemented within *Sim4Life* are not available, but details on modifications to the bioheat equation to allow variable blood temperature have been published by others.^21–23^ The blood volume used was 324 mL, which is typical of a 3.6-kg term neonate a few days after birth.^24^

The thermal conductivity and thermal diffusivity of water are higher than those of other constituents of biological tissue, and so the thermal properties of neonatal tissues may also be expected to reflect the higher water content relative to adult tissues. Models for inferring these properties from water content do exist, but require precise knowledge of the thermal properties of tissue proteins and have proven difficult to validate experimentally.^25^ In light of these difficulties, adult values for tissue-specific thermal properties taken from a database^19^ were assumed in the present study. This is expected to be conservative, as the neonatal conductivity and heat capacity would both be expected to increase with greater water content, leading to lower temperatures.

Thermal modeling of adults exposed to RF fields often includes temperature dependence of tissue perfusion.^26^ However, because neonates’ thermal regulation capability is underdeveloped, in this study temperature-independent tissue perfusion was assumed. This provides conservative estimates, as increased perfusion in response to local temperature increases acts to reduce local temperatures in the bioheat model.

To address the fact that neonates require thermal insulation to maintain body temperature, the neonate A model was constructed with an insulating “blanket” layer, assumed to have the thermal properties of wool. Heat transfer between the outer surface of the model and environment is determined by heat transfer coefficient *h* (units W m^−2^ K^−1^) in *Sim4Life*. Note that the model’s “outer surface” is either exposed skin or the outer blanket surface, but for a given model the entire surface is given a single value of *h*. This parameter, along with time-dependent core temperature, was tuned to match literature reports that suggest that passive cooling of termaged neonates results in core temperatures within the range of 33°C-34°C within 2-3 hours.^27,28^

An additional complexity is that the simulations must first be run for some period to obtain a physically realistic temperature distribution, as the starting point has a uniform temperature in all tissues (37°C) with a 22°C background temperature. We found this to be particularly important for thermal simulations involving a variable core temperature that, if activated from an unrealistic initial condition, gives unreliable results. An initial simulation starting with a uniform temperature in all tissues (37°C), background temperature of 22°C, *h* = 8 W m^−2^ K^−1^, and time-dependent core temperature deactivated, was run for 60 minutes before all simulations. This resulted in a maximum temperature of 37.6°C in the heart and a mean skin temperature of 33.8°C for neonate A, the latter in general agreement with results in the literature.^29,30^

The neonate was simulated with and without the blanket, referred to as “insulated” and “uninsulated,” respectively. Initial investigations showed that setting *h* = 11 W m^−2^ K^−1^ with time-dependent core temperature activated and no RF exposure resulted in an essentially stable core temperature for the insulated case. Use of the same conditions for the uninsulated case resulted in a fall in core temperature to 34.3°C after 3 hours, in general agreement with the passive cooling results from the literature.^27,28^ The same value of *h* was used for insulated and uninsulated cases; the insulating effect of the blanket is accounted for by the low thermal conductivity of the blanket layer.

A thermal simulation involving Duke was also run for comparison using the same process to generate the starting temperature distribution, with *h* = 11 W m^−2^ K^−1^ thereafter. Temperature-dependent perfusion for skin, muscle, fat, and brain tissues, as described in Murbach et al,^31^ was also activated after the initial period. Time-dependent core tem-perature was not used for the adult, as a healthy adult would regulate their temperature actively, using mechanisms not well-captured by the simple model.

For RF exposure cases, the power level was set to the maximum level allowed within IEC guidelines for SAR. As will be outlined in section 3, we found that the first SAR limit^5^ to be reached for all models (including the adult) was head average SAR ≤ 3.2W kg^−1^, although in many cases this was very close to psSAR_10g_ ≤ 10 W kg^−1^. Thermal simulations were run at an average power level of 8W for neonate A and 21.3 W for the adult.

### 2.6 Additional EM simulations

To investigate position sensitivity, calculations were repeated for the neonate models (neonatal dielectric properties) shifted by ±25 mm in transverse directions, and −50 mm to +50 mm in the superior–inferior direction.

The effect of the size of the object on the B_1_^+^ distribution was investigated by simulating the 7T head coil loaded with uniform cylinders of various diameters ranging from 80 mm to 200 mm (length = 250 mm). These cylinders were given the dielectric properties of (adult) muscle.

To compare the results for the 7T head coil with previous findings using a larger body coil at 3 T,^7^ simulations were repeated after retuning the head coil model used in this work down to 127 MHz and removing the RF shield, which compromised performance at the lower frequency. Appropriate dielectric properties were used for this frequency, including age-adjusted corrections as previously published in Malik et al.^7^

### 2.7 Model availability

The neonate A model created as part of this study is available for download at https://doi.org/10.5281/zenodo.4535237.

## 3 RESULTS

### 3.1 Electromagnetic simulations

**Figure 2:**
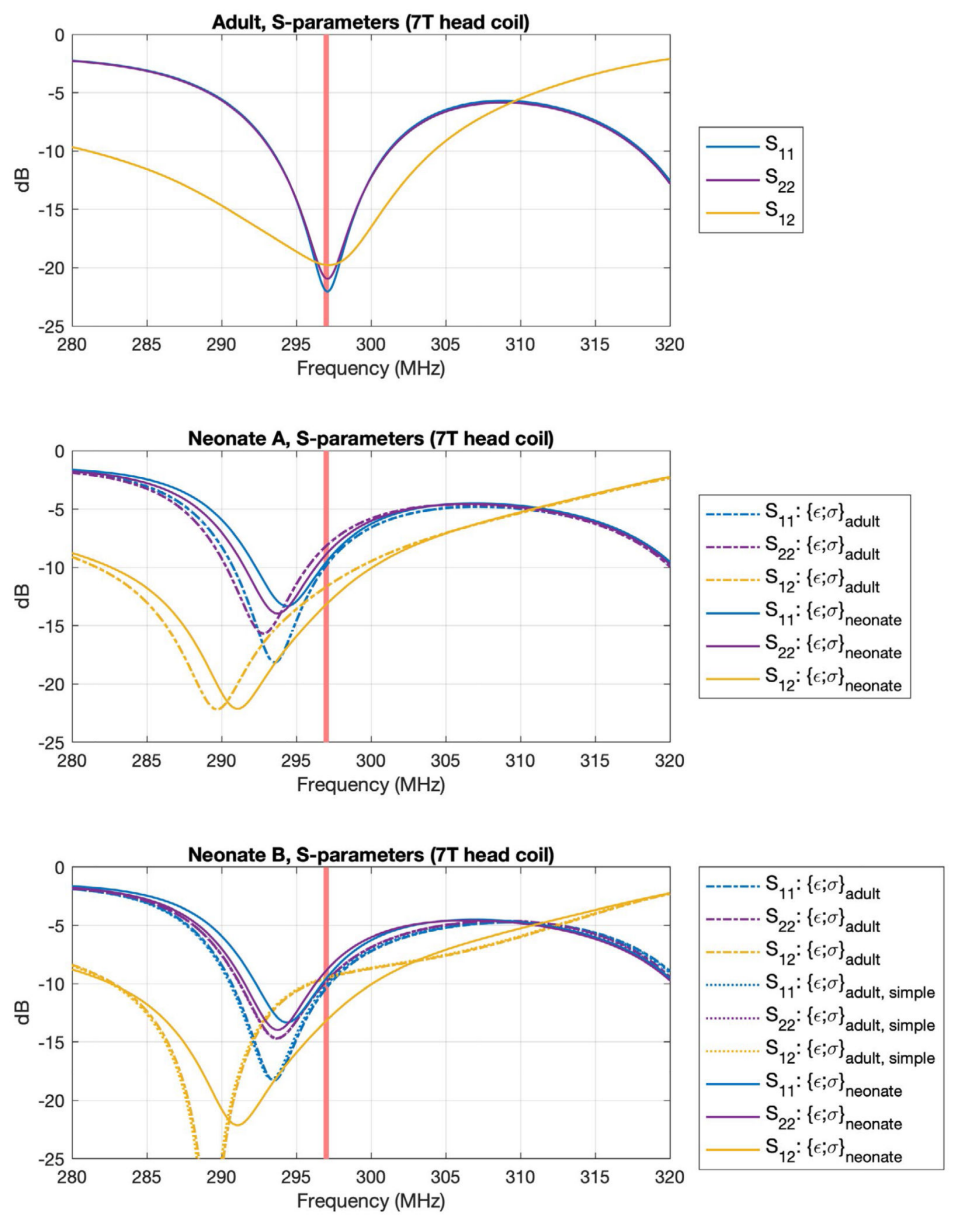
S-parameters of the simulated birdcage coil (driven at two ports) for each of the voxel models simulated. The coil was tuned and matched at 297 MHz (indicated by red line) using the adult voxel model, and generally good matching can be seen for this model (S_nn_≤ 20 dB). We observe some shifting of the resonance frequency when loaded with neonate models, but still obtain acceptable return loss. Neonates were simulated both with adult ({ε;σ}_*adult*_) and neonate-appropriate ({ε;σ}_*neonate*_) dielectric properties. Neonate B was also simulated with a simplified tissue segmentation ({ε;σ}_*adult,simple*_) (details in text)

Table 1 summarizes the results of EM simulations for each model, and S-parameters of the coil with different loads are shown in Figure 2. The neonate models change the loading, leading to a shift in the resonance frequency. The proportion of power absorbed by the neonate was less than the adult; in addition to higher reflected power, we observed higher radiated power for neonates (~22% for neonate A, ~27% for neonate B, ~7% for Duke).

The SAR experienced by the neonates per watt of (total) input power is higher than the adult. Both head average and the psSAR_10g_ values are in the order of 2 to 3 times greater than the adult case. The very large difference between whole-body exposure levels is due to the fact that a much smaller fraction of the adult’s body is exposed to the fields from the head coil when compared with the neonate.

However, less power may generally be required for imaging the neonate, so the SAR values were also normalized to achieve 1-μT B_1_^+^ in a central axial slice. In this case, the relative differences between adult and neonate are smaller. When using adult dielectric properties, the psSAR_10g_ values for the two neonate models are 26% and 18% greater than the adult, respectively, while head averaged values are 62% and 17% greater, respectively.

The largest differences occur when age-adjusted dielectric properties are used for the neonate models. In this case, the SAR normalized to B_1_^+^ is 85%-100% greater than the adult for neonate A (depending on whether head average or peak spatial values are considered) and 75%-85% greater for neonate B. These large differences occur primarily because B+ per ✓W is reduced when neonatal dielectric properties are used.

**Figure 3:**
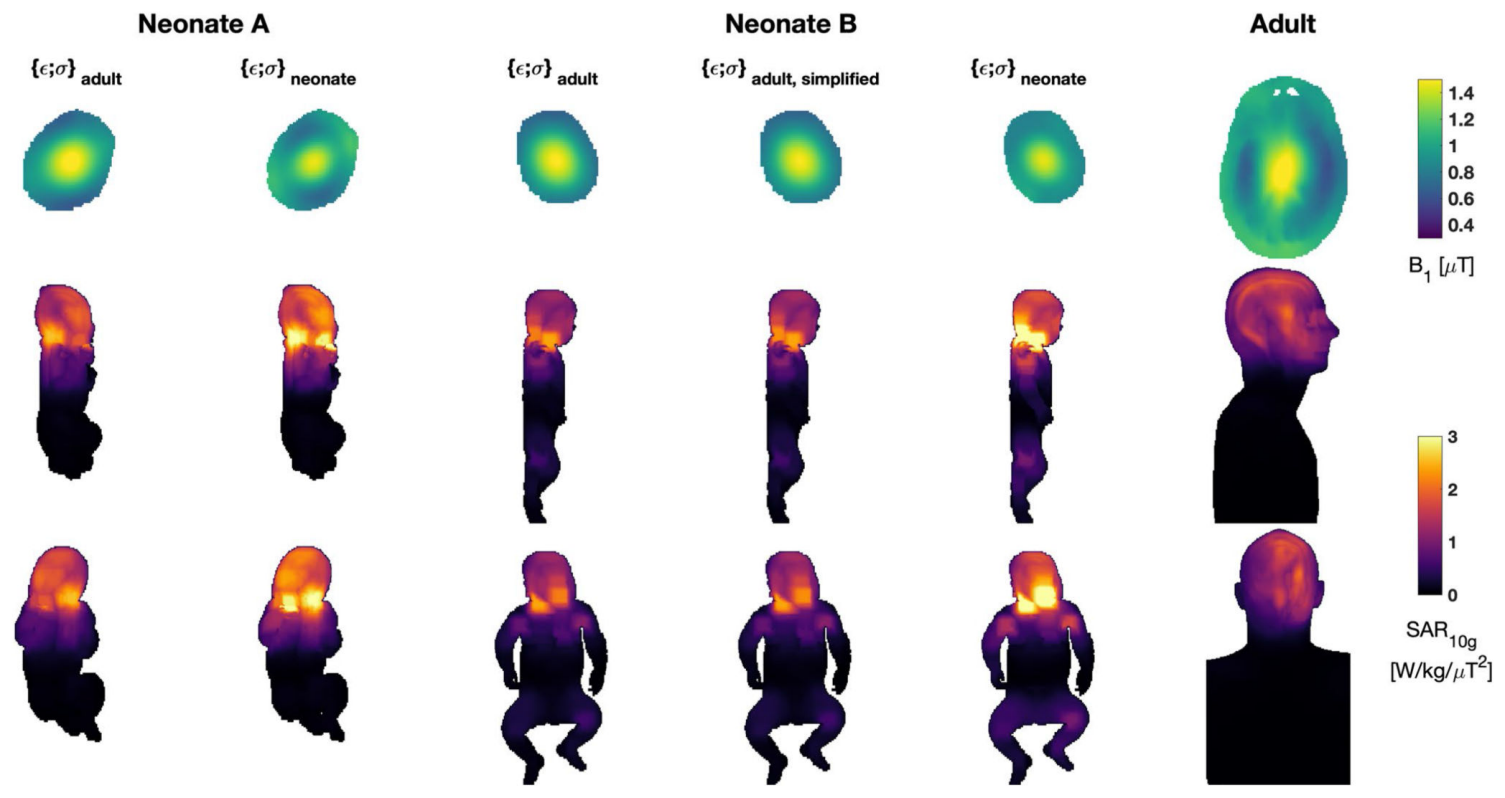
The B_1_^+^ and 10*g* specific absorption rate (SAR_10g_) for all neonate models and Duke. The neonate models were simulated using both adult and neonate specific (age-adjusted) dielectric properties. Neonate B was also simulated using a simplified tissue segmentation to match the lower number of separate tissue classes in model A. Models are all depicted at the same spatial scale. Top row: B_1_^+^ distributions in central axial plane for mean B_1_^+^ = 1 μT; this slice is the one used to normalize the SAR distributions also reported in Table 1. Middle/bottom rows: SAR_10g_ distributions as maximum projections in sagittal/coronal views, respectively

Figure 3 depicts the same results, also showing the spatial distributions. The location of local SAR maxima is similar for both neonate models, occurring in the posterior part of the neck. The presence of the hand near the face for neonate A also leads to higher SAR close to the face in this model. When adult dielectric properties are used, the effect is small; however, the version using neonatal dielectric properties has a changed field distribution with a more pronounced “hotspot” on the face and the global SAR maximum occurring in the fingers of the right hand.

Neonate B was also modeled using a simplified tissue segmentation, to more closely match the relatively crude segmentation made for neonate A. Table 1 and Figure 3 both suggest that the difference between this and the fully segmented model is small.

**Figure 4:**
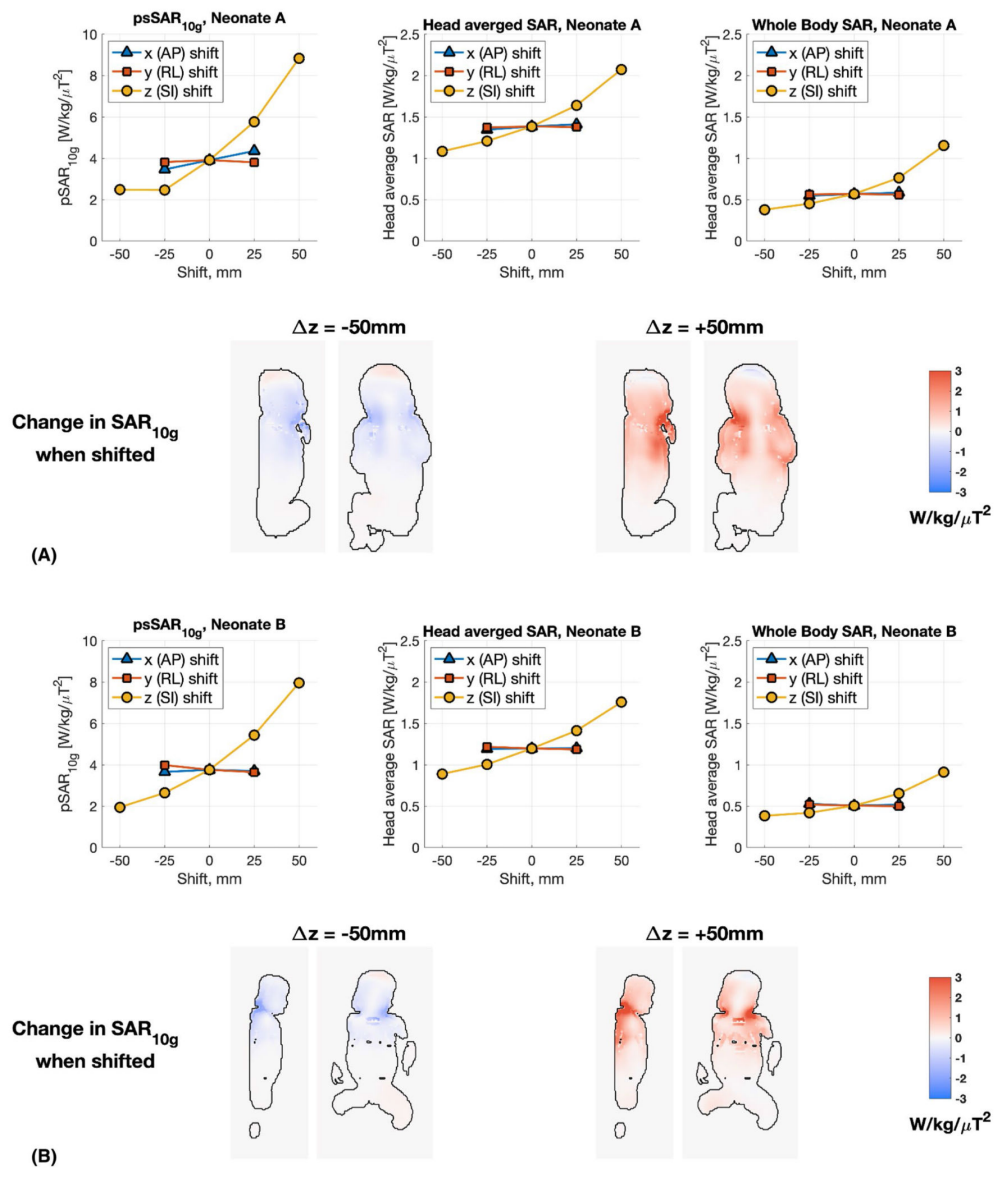
Summary of changes in simulated SAR when the neonate models are shifted inside the coil (A, neonate A; B, neonate B; both cases using neonatal dielectric properties). In each case, the SAR values are normalized by the average B_1_^+^ within the same anatomic slice (shown in Figure 3) (ie, the slice position is shifted as the model is shifted). Inset images show the representative slices highlighting changes in SAR_10g_ with respect to no spatial shift. Abbreviations: AP, anterior–posterior; RL, right–left; SI, superior–inferior

Figure 4 summarizes the EM simulation results for shifting the neonate models within the head coil. Similar trends were seen for both models. The SAR values are largely unaffected by small shifts in the transverse directions, with the only exception being anterior–posterior shifts for neonate A, which resulted in variations of ±10% in psSAR_10g_ (head average and whole-body average changed by <3%). Superior– inferior shifts resulted in much larger SAR variability, and again both models showed similar results; psSAR_10g_ increased by 100%-125% when the neonate is shifted by +50 mm (ie, further into the coil) and reduced by 40%-50% when shifted −50 mm (ie, out of the coil). The data presented are normalized to B_1_^+^ within the same anatomic slice (ie, the slice shifts as the neonate shifts), to simulate imaging of the same anatomy with an altered positioning of the subject.

**Figure 5:**
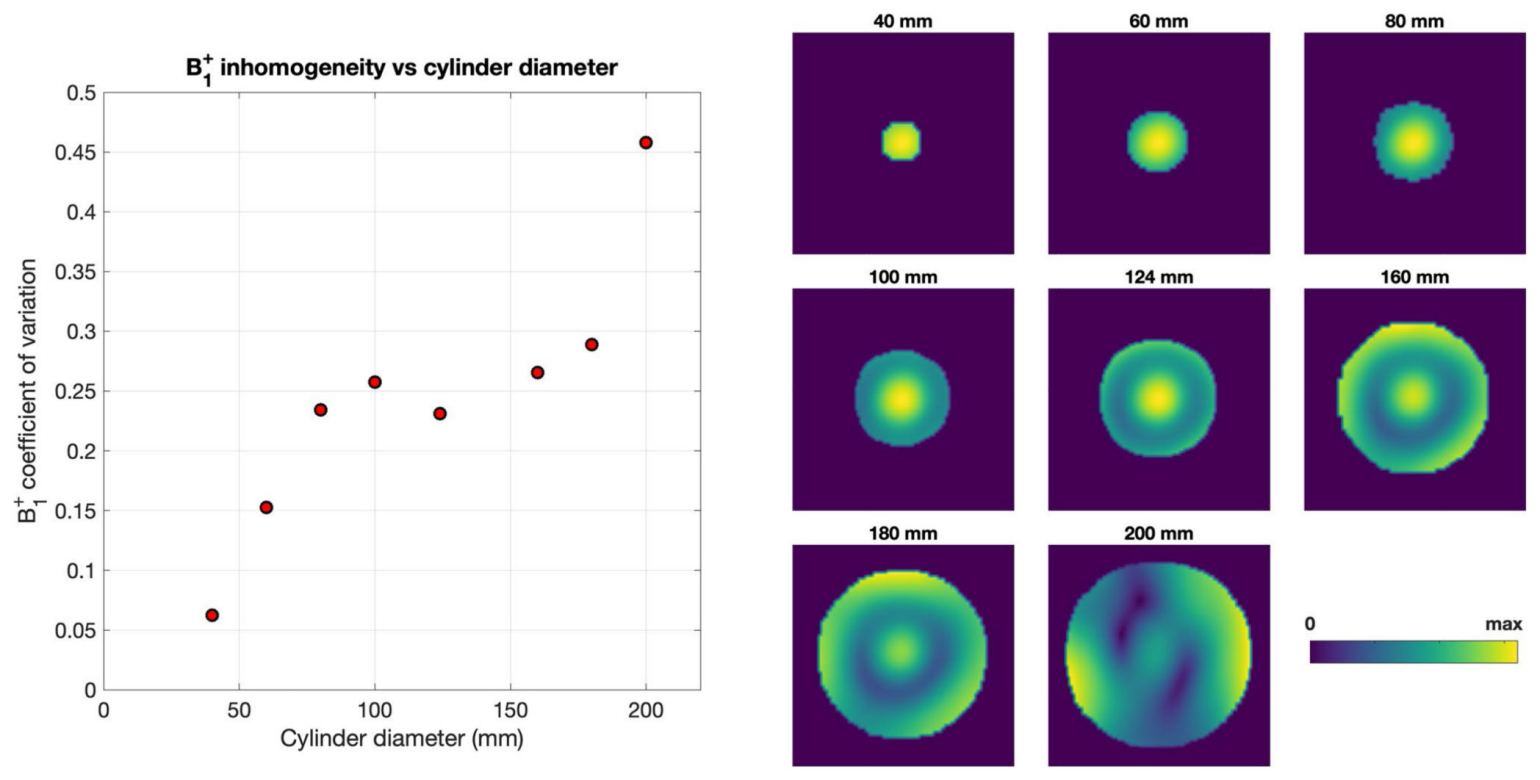
Simulated B_1_^+^ distributions and homogeneity measure (coefficient of variation) for cylinders of different diameter within the 7T head coil. Cylinders had the same dielectric properties as muscle. B_1_^+^ distributions are shown at the same relative scales (ie, between maximum and minimum values) to highlight the homogeneity of the pattern rather than the absolute value. As expected, the homogeneity worsens as the diameter increases; however, it remains within a similar range between the diameters of 80 mm and 180 mm

Another observation from Figure 3 is that the B_1_^+^ inhomogeneity in neonates is predicted to be similar to adults, as quantified by coefficient of variation in Table 1. To explore this further, Figure 5 shows simulated B_1_^+^ within cylinders of varying diameter within the same coil. The B_1_^+^ coefficient of variation generally increases with diameter but remains fairly flat in the 80-180-mm range. Mean head diameters of neonates A and B were approximately 100 mm, while the adult (Duke) is approximately 180 mm.

To explore the frequency dependence of these data, the same coil was tuned to 127 MHz, with results summarized in Table 2 and field distributions given in Supporting Information Figure S1, S-parameters in Supporting Information Figure S2, and a comparison of the B_1_^+^ field dependence on phase offset between ports in Supporting Information Figure S3.

### 3.2 Thermal simulations

**Figure 6:**
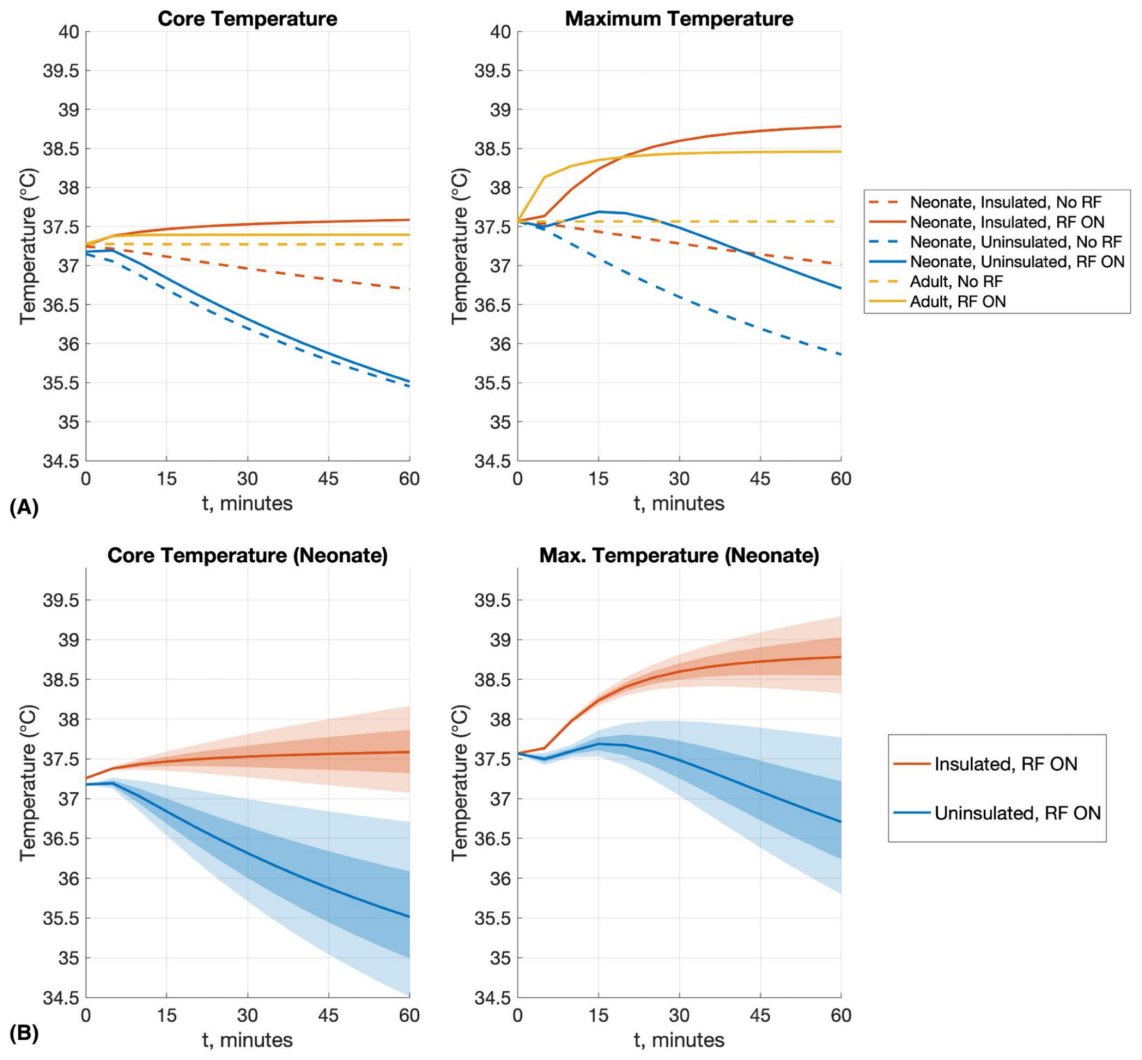
A, Summary temperature results for neonate A (neonatal properties) with and without thermal insulation (“blanket”), and the Duke adult model for comparison. Simulations were run for 60 minutes before this, to establish thermal equilibrium (details in text). For all scenarios, the simulations were run such that head-average SAR = 3.2 W/kg, as this was the most limiting in all cases. B, Because the heat transfer coefficient (*h*) for neonates is not well understood, simulations were repeated by changing *h* by ±10% (darker shading) and ±20% (lighter shading)

Figure 6A plots the core and maximum temperature for models including and excluding an insulating blanket. The core temperature was estimated by averaging the 3D temperature data within the core regions of the heart and brain. When the neonate is insulated (with blanket), the core temperature falls by about 0.5°C over 1 hour when not exposed to RF. With RF exposure, the core temperature of the insulated neonate increases by about 0.3°C over an hour, which is within the 0.5°C IEC guideline limit.^5^ Moreover, the maximum temperature does not exceed the 39°C absolute limit within this period. In the case of the uninsulated model, the core temperature falls to approximately 35.5°C after 1 hour, suggesting mild hypothermia. The application of RF energy initially arrests the fall, but after 5 minutes the core temperature continues to fall, reaching a similar level to the no-RF condition. In this case, the maximum temperature in the presence of RF falls from 37.6°C to 36.7°C after 1 hour. Simulations including RF exposure were repeated, changing the heat transfer coefficient *h* by ±20%, as shown in Figure 6B.

**Figure 7:**
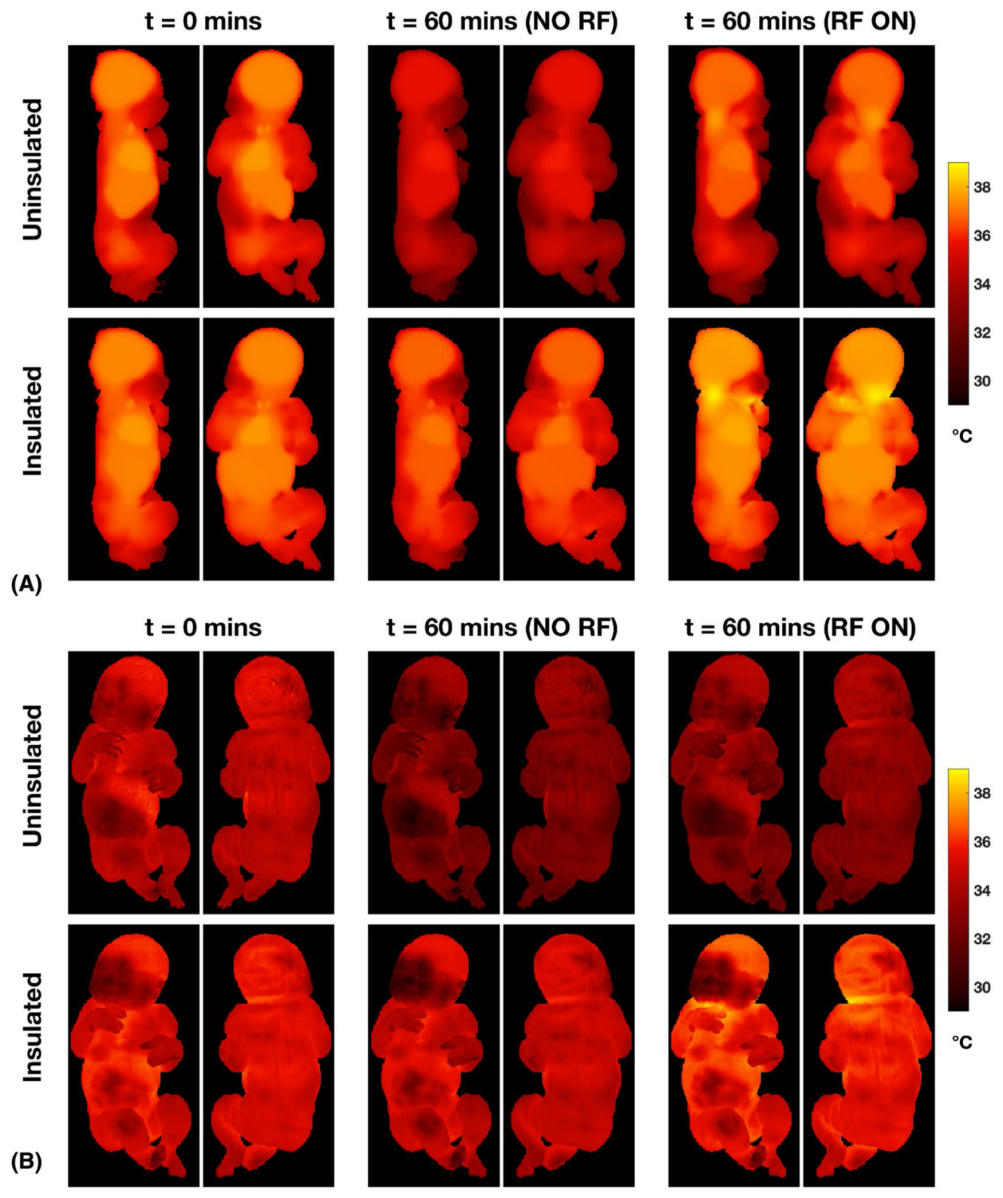
Temperature distributions for neonate A with and without thermal insulation (blanket). A, Maximum intensity projections. B, Surface temperature distributions. The starting time (*t* = 0) was reached after 60 minutes of simulation to reach a stable starting condition. Temperature distributions after a further 60 minutes without (middle columns) or with (right columns) RF exposure are shown. Without thermal insulation or RF exposure, the neonate cools significantly. When insulated and with 60 minutes of RF exposure, higher local temperatures are reached in the posterior part of the neck, and the face/hand where these are close together, but do not exceed 39°C

Figure 7 shows the maximum temperature projections (Figure 7A) and surface temperature distributions (Figure 7B) for the different scenarios. The maximum temperature for the insulated neonate occurs at the back of the neck. The surface-temperature distributions show that the face, which is not covered by the blanket in this model, remains cooler than the rest of the skin, as would be expected. Equivalent figures for the adult model are shown in Supporting Information Figure S4 for comparison.

## 4 DISCUSSION

This safety assessment for neonatal MRI at 7 T considered EM and thermal simulations of RF exposure at 297 MHz within a single-channel head coil (birdcage design). We examined two different neonate models, one of which was created for this study, alongside a standard adult model (“Duke”). Specific to neonatal imaging, we also considered the effect of adjusting dielectric properties for increased water content of neonatal tissues. Thermal simulations were also adapted to neonates by including thermal insulation, as neonates would in reality become hypothermic without this.

The head-averaged SAR and psSAR_10g_ values normalized to absorbed power are higher in the neonates than the adult by factors ranging from approximately 3.9-4.1 and 3.2-4.1, respectively. If normalized instead to net-forward power (forward-reflected), these ratios become 2.4-3.0 and 2.4-2.8, respectively. When normalized to the square of mean B_1_^+^ in the central axial slice, these differences are reduced further with ratios of neonate/adult for head-averaged SAR in the range of 1.2-1.6 and psSAR_10g_ in the range of 1.2-1.3, if the neonates are assumed to have the same dielectric properties as the adult. The use of dielectric properties that account for the higher water content of neonatal tissues leads to small changes in SAR per input power, but significantly reduces the mean B+ achieved per -\/*w*. The result is that the SAR per square B_1_^+^ substantially increases (head-averaged SAR is 1.75-2.0 times larger in the neonate models compared with the adult, and psSAR_10g_ is 1.8-1.9 times greater).

The SAR values were comparable for the two models (A and B) with local maxima in the neck region. In addition, neonate A has a SAR maximum caused by proximity of the hand to the face; this model was constructed from neonatal (3T) MR data, and this posture is not uncommon for scanning of infants. Neonate A used a much less detailed tissue segmentation than neonate B (13 tissue types compared with 31). A simplified version of neonate B was constructed by assigning multiple tissue types to a generic “connective tissue” category. This was a simplistic approach; a more physically realistic method would assign properties of neighboring tissues. Nevertheless, the differences caused were on the order of 1%-2%, suggesting that this would not be a major cause of error. Others have reported a similar robustness of EM simulations to reduced segmentation quality.^32,33^

A recent study from Annink et al^6^ examined scanning of neonates at 7 T using a similar head coil, with the 2-month-old model “Charlie” (Zurich MedTech). Neonate B is derived from the same source as Charlie but has been scaled down to closer in size to the median neonate. Our study found psSAR_10g_ in the same range but larger (2.41 W kg^−1^ μT^−2^ compared with 1.48 W kg^−1^ μT^−2^) when using adult dielectric properties, and significantly larger if age-adjusted dielectric properties are used instead (which was not considered in Annink et al^6^). In contrast to the neonate/adult SAR ratios quoted previously, which were all greater than 1, Annink et al^6^ found a ratio of 0.47 for psSAR_10g_ and 0.63 for head-average SAR. We also found significantly greater head- averaged SAR values in all models than in Annink et al^6^; the ratio of peak to head-averaged SAR is in the range of 2.3-3.1 across all models in our study (3.0 for Duke), but is 5.1-6.6 for similar models in Annink et al^6^ (6.6 for Duke).

This discrepancy suggests that there is a systematic difference between the coil models used in the two studies. A study by Restivo et al,^34^ using a similar (slightly shorter) 7T head coil,^35^ reported similar psSAR_10g_ of approximately 2.6 W kg^−1^ μT^−2^ for the Duke model, compared with 2.05 W kg^−1^ μT^−2^ in this study. They also observed the ratio of peak to head-averaged SAR was in the range of 2.9-3.7 for four “patient based” models derived by blending the Duke model with patient data. Van Lier et al^35^ reported a ratio of 3.8 for Duke. Annink et al also considered shifting position of the neonate, and their results broadly agree with ours, showing an approximate doubling of psSAR_10g_ if the neonate is moved +50 mm superior, for example. The large variability of SAR with superior–inferior shifts can be understood by the changing exposure of the whole of the neonate’s body to the fields, and movement of the worst-case SAR regions (back of neck, and hand near face for neonate A) toward or away from the center of the coil (Figure 4).

We previously investigated neonatal SAR exposure at lower field strengths^7^ and generally found the SAR experienced by neonates for matched imaging conditions (equivalent mean square B_1_^+^) was always less than adults by a factor of 3 or 4. The present study for 7T imaging found that the SAR experienced by neonates is generally higher than an adult by approximately a factor of 2 for equivalent mean square B_1_^+^. In addition to the different resonance frequency, the previous study considered a large body resonator and had the adult positioned heart-centered in the body coil, making a direct comparison difficult with the present work. To bridge between these results, we repeated our simulations with head coil tuned to 127 MHz. These simulations should be treated with caution, as the device was not designed for operation at 127 MHz, and aside from retuning, it was not optimized (these results are for comparison only and should not be used to judge the performance of a well-designed device at this frequency). Results (Table 2) show it was poorly matched for a neonate. Moreover, Supporting Information Figure S2 shows that the mode structure of the coil is as expected, and Supporting Information Figure S3 shows plausible B_1_^+^ fields when driven in circular polarization and anti–circular polarization modes in both adult and neonate. Hence, we focus on the SAR normalized to B_1_^+^. The 7T head coil gave a ps- SAR_10g_ of approximately 3.8 W kg^−1^ μT^−2^ for the neonate (age-adjusted dielectric properties) and approximately 2 W kg^−1^ μT^−2^ for the adult. The head coil at 127 MHz gave a psSAR_10g_ of about 0.6-0.7 W kg^−1^ μT^−2^ for the neonates with age-adjusted properties and about 0.8 W kg^−1^ μT^−2^ for the adult, suggesting that at 3 T the SAR experienced by the neonate is comparable with the adult, but that this increases more quickly in neonates with frequency, potentially because of the smaller size and/or dielectric property differences.

Another surprising result is the prediction that B_1_^+^ inhomogeneity would be similar between neonates and adults, when it might be expected that smaller head size would lead to more uniform fields. Table 1 indicates that the coefficient of variation of B_1_^+^ is in the range of 0.2 for all models. To investigate this further, we simulated B_1_^+^ in cylinders of different diameters (Figure 5): The coefficient of variation increases with cylinder diameter but plateaus in the 80-180-mm range, which encompasses the neonate and adult models. This simple analysis does not account for shape-based variation, but does support the observations made in human models.

Previous MR safety studies of neonates did not consider thermal modeling, which for neonates is complicated by their underdeveloped thermal regulation ability, possible use of sedation, high surface to volume ratio (ie, high dependence on thermal boundary conditions), and poorly defined tissue thermal properties. Neonates can only maintain body temperature in an appropriately controlled, humidified, and temperatureneutral environment,^36,37^ and risk becoming hypothermic while in the normal MR environment.^38^ Wheldon^39^ studied neonatal heat loss using a heated manikin and reported that coefficients for both radiation (*h_r_*) and convection (*h_c_*) were dependent on posture, with *h_r_* increasing from 3.1-4.9 W m^−2^ K^−1^ and *h_c_* from 4.0-5.4 W m^−2^ K^−1^ as posture changed from fetal to spread-eagled. Summing these coefficients implies an overall loss coefficient in the range of 7-11 W m^−2^ K^−1^, although this excludes heat loss through evaporation, which would increase these further. Our thermal simulations do not explicitly include all of these mechanisms, and were instead tuned to give reasonable behavior in the absence of RF exposure. For the uninsulated neonate, the time taken to reach a core temperature of 34.3°C was 3 hours, which is within 1 SD of the mean time (2.67 ± 1 hour) reported in Kendall et al,^28^ which also reported a wide range of times (1.25-5.5 hours) taken to reach a similar core temperature.

The thermal models investigated in this study predict that a naked neonate becomes hypothermic in an environment of 22°C, even when including RF exposure to recommended SAR limits. Moreover, when the same neonate is insulated and thermally stable, exposure to the same RF results in elevated core and local temperatures that remain compliant with recommended limits up to 60 minutes. Because the overall heat transfer coefficient is not well known, we investigated a spread of *h* and found that core-temperature increase exceeds the recommended limit of 0.5°C^5^ after 25 or 40 minutes, and maximum temperature reaches the IEC limit^5^ of 39°C after 40 or 60 minutes, when *h* is reduced by 20% or 10%, respectively.

Although simulated local temperature hotspots are located in the same place as the psSAR_10g_ hotspots (Figure 7), temperature increases are widespread. This is because the neonate is small and has a large degree of exposure. The coil acts somewhat like a whole-body coil for the neonate, whereas it is local for an adult. Indeed, the whole-body SAR, while never the limiting case for a neonate, is much larger than an adult (~0.5 W kg^−1^ μT^−2^ compared with ~0.05 W kg^−1^ μT^−2^). For example, at the head-average SAR limit (3.2 W kg^−1^) for neonate A (neonatal dielectric properties), the whole-body SAR would be 1.3 W kg^−1^ and psSAR_10g_ would be 8.8 W kg^−1^. Hence, it would seem important to consider systemic as well as local heating effects.

### 4.1 Uncertainties

The RF coil model studied was tuned to an adult load, as this is the more likely configuration for a commercial coil intended for adults. Frequency shifts of about 1% were observed when loaded with a neonate; others have reported birdcage coils with similar load sensitivity.^40,41^ However, the current patterns could differ from a device specifically tuned to a neonate, which may change the results. The ventricles are visible in our simulated B_1_^+^ maps for the adult (Figure 2), which is not necessarily expected, although it has previously been reported as a potential conductivity-related effect.^42^

We have found that using age-adjusted dielectric properties results in significantly larger predicted SAR when normalized to achieved B_1_^+^. Although to the best of our knowledge there are no reports of measured human neonatal dielectric properties in the public domain, it should not be considered surprising that these would differ significantly from adults; it is well known that NMR relaxation times change significantly over the first year of life, and these are related to changes in water content.^43^ Most of the data regarding neonatal dielectric properties are derived from rats, mice, rabbits, and pigs.^44^ Reports of the water content of rat tissues indicate a reduction with increasing age,^45,46^ and comparisons have been made with data from human tissues.^47^

The uncertainties associated with measurements of tissue permittivity and conductivity are typically 6%-10%, depending on water content.^48^ According to Wells et al,^49^ the uncertainty in predicting of water content in neonates from anthropometry is approximately 16%. Local SAR_10g_ is dependent on local geometry, so these values are subject to greater uncertainty, especially when attempting to represent a population using a small number of models. In the case of neonates, there is additional uncertainty due to the changing composition of the subject, such as the amount of adipose tissue. It is difficult to assess this uncertainty, but it is likely to be about 10%.^50^

Similarly, the results of this initial thermal model of a neonate within the MR environment should be interpreted with care. This study used adult values for tissue thermal properties, as no reliable alternatives are available. Heat loss arises through several mechanisms, not all of which are accounted for in the present study. Other complicating factors include the underdeveloped thermal regulation in neonates and the effect that posture has on heat loss and SAR. Although sophisticated numerical models have been developed to simulate heat and mass-transfer mechanisms for other applications in neonatology such as in incubators and for radiant warmers,^51^ their application to the MR environment is hampered by the need to accurately account for a spatially inhomogeneous SAR distribution as well as the factors referred to previously. Accurate modeling of the unique thermal conditions for neonates is challenging and is ongoing.

## 5 CONCLUSIONS

Unlike at lower field strengths, our findings suggest that 7T MRI of neonates is not intrinsically safer than for larger subjects, from an RF heating perspective. The MR scanners differ in their method for controlling SAR exposure, some computing limits based on net power and others based on B_1_^+^, for example. For limits based on measured power, our results suggest that neonatal SAR is approximately 2.4-3 times greater than the adult when normalized to net forward power (forward-reflected) or 4 times greater when normalized to absorbed power. Higher B_1_^+^ efficiency compensates for this to a degree: The SAR per square B_1_^+^ approximately doubles compared with the adult, suggesting that the RMS B_1_^+^ available for imaging neonates should be reduced by ✓^ (a 30% reduction). This does not include any additional safety margin for uncertainty in neonatal tissue properties, intersubject or position variability. Determining safety margins is somewhat beyond the scope of the work presented so far. Intersubject variability is a problem for all SAR studies, which tend to look at only a small number of models; our data on two models showed approximately similar behavior on both. Position sensitivity has recently been shown to be a major issue for SAR calculations.^52^ We found that superior–inferior shifts could lead to a doubling of SAR; in practice, careful positioning might be used to mitigate risk and avoid overly conservative safety margins.

A recent safety evaluation for neonatal 7T MRI^6^ concluded that SAR limits for neonates (based on B_1_^+^) are similar to adults. Although some of our results agree with that study, overall our data do not support this conclusion, particularly if age-adjusted dielectric properties are considered.

Finally, this study also considered thermal models, as neonates are usually thermally insulated when scanned, to avoid hypothermia. We found that temperatures should not exceed IEC limits if SAR limits are respected, but that this is dependent on the heat transfer coefficient with the environment (*h*); reduction of 20% from the value used here would see thermal limits exceeded within 25 minutes. Systemic as well as local temperature changes are likely to be important; hence, active monitoring and management of body temperature would seem necessary.

## Supplementary Material

Supporting information

## Figures and Tables

**Table 1:** Summary of EM simulation results

		Neonate A		Neonate B			Duke
Dielectric properties		Adult	Neonatal	Adult	Adult, simplified	Neonatal	Adult
Reflected power (%)		13	11	17	17	16	0.4
Absorbed power (%)		58	59	47	47	52	88
Mean B_1_^+^ for 1 W input (μT/✓W)		0.61	0.53	0.62	0.61	0.53	0.47
B_1_^+^ coefficient of variation		0.23	0.17	0.22	0.22	0.16	0.19
SAR per input power(W/kg/W)	Head average	0.41	0.40	0.32	0.31	0.35	0.15
	Whole-body average	0.17	0.16	0.13	0.13	0.15	0.01
	psSAR_10g_	0.95	1.10^*^ (0.92)	0.94	0.91	1.08	0.45
SAR per mean B_1_^+^ (W/	Head average	1.12	1.39	0.81	0.83	1.21	0.69
kg/μT^2^)	Whole-body average	0.45	0.57	0.35	0.35	0.51	0.05
	psSAR_10g_	2.58	3.84^*^ (3.21)	2.41	2.41	3.78	2.05

*Note:* The SAR is given both per (total) input power and per mean B_1_^+^, with 100% duty cycle. The mean B_1_^+^ was calculated as the average over a central axial slice, depicted in Figure 3. Data for the brain-centered Duke model are included for comparison. In all but one case, the peak spatial 10*g* SAR (psSAR_10g_) was in the head; the asterisk indicates the exception, in which case the number in brackets gives the maximum in the head.

**Table 2:** Summary of simulation results for the head coil tuned down to 127 MHz (3 T)

		Neonate A		Neonate B		Duke
Dielectric properties		Adult	Neonatal	Adult	Neonatal	Adult	
Mean B_1_^+^ for 1 W input (μT/✓W)		0.85	0.83	0.86	0.86	0.71
Absorbed power (%)		17	25	12	18	66
SAR per input power (W/kg/W)	Head average	0.12	0.17	0.09	0.13	0.13
	Whole-body average	0.04	0.06	0.03	0.05	0.01
	psSAR_10g_	0.33	0.48	0.28	0.47	0.41
SAR per mean	Head average	0.17	0.24	0.12	0.18	0.26
B_1_^+^ (W/kg/μT^2^)	Whole-body average	0.06	0.09	0.04	0.06	0.02
	psSAR_10g_	0.45	0.69	0.39	0.63	0.81

*Note:* The device was only retuned (and the RF shield removed), but otherwise not optimized. It is clear that the coil is not well matched, so instead we focused on the SAR normalized to B_1_^+^ in further analysis. The resulting B_1_^+^ and SAR distributions are shown in Supporting Information Figure S1. The psSAR_10g_ per square B_1_^+^ is smaller for the neonate models if the same dielectric properties are used, but becomes comparable to the adult if age-adjusted properties are used.
