# Supplementary material for: Specific absorption rate and temperature in neonate models resulting from exposure to a 7T head coil
Source: Magn Reson Med. Author manuscript; Available in PMC 2023 Jul 12. (PMC7614747; doi:10.1002/mrm.28784)
Supplement: Supporting information [file EMS177409-supplement-Supporting_information.docx]

| **Model** | **Mass** | | | | | **Length** | | | | |
| --- | --- | --- | --- | --- | --- | --- | --- | --- | --- | --- |
|  | Mass  (kg) | Percentile  mass for age | | z-score | | Length  (m) | Percentiles  length for age | | z-score | |
|  |  | Males | Females | Males | Females |  | Males | Females | Males | Females |
| Neonate A | 3.62 | 71st | 79th | 0.55 | 0.82 | 0.50 | 52nd | 68th | 0.06 | 0.46 |
| Neonate B | 3.57 | 67th | 76th | 0.45 | 0.71 | 0.49 | 32nd | 47th | -0.47 | -0.08 |

**Supporting Information Table S1**. Mass and length of the 2 neonate models, new born percentiles, and z-scores (relative to median values) according to WHO (ref 14 in paper). The z-score is the deviation of an individual's value from the median value of the population, divided by the standard deviation of the population. Note that the mass values were estimated by combining voxel model volumes with tissue density data from the Sim4Life database. The density data are not corrected for higher water content of neonatal tissue and may hence slightly overestimate the mass, since tissue is in general denser than water. The recorded birth mass of the neonate whose images were used to make Neonate model A was 3.50 kg.

| **Tissue** | **Permittivity ratio** | **Neonate**  **permittivity** | **Conductivity ratio** | **Neonate conductivity (S/m)** | **Neonate** | | |
| --- | --- | --- | --- | --- | --- | --- | --- |
|  |  |  |  |  | **A** | **B** | **B_simplified_** |
| Adrenals | 1.3 | 80.91 | 1.5 | 1.00 | 🗶 | ✓ | 🗶 |
| Bone | 2.2 | 29.58 | 3.83 | 0.32 | ✓ | ✓ | ✓ |
| Bladder wall | 1.3 | 26.13 | 1.5 | 0.47 | 🗶 | ✓ | 🗶 |
| Bladder contents | 1.0 | 49.95 | 1.0 | 1.75 | 🗶 | ✓ | 🗶 |
| Blood | 1.0 | 65.71 | 1.0 | 1.32 | ✓ | 🗶 | 🗶 |
| Brain | 1.39 | 83.22 | 1.68 | 1.62 | ✓ | ✓ | ✓ |
| Breast | 1.3 | 81.21 | 1.5 | 1.28 | 🗶 | ✓ | ✓ |
| Connective tissue | 1.3 | 62.41 | 1.5 | 0.80 | 🗶 | ✓ | ✓ |
| Eye humor | 1.0 | 69.02 | 1.0 | 1.52 | ✓ | ✓ | ✓ |
| Eye lens | 1.3 | 49.90 | 1.5 | 0.53 | 🗶 | ✓ | 🗶 |
| Fat | 1.3 | 15.27 | 1.5 | 0.11 | ✓ | 🗶 | 🗶 |
| Gall bladder wall | 1.3 | 81.86 | 1.5 | 1.67 | 🗶 | ✓ | 🗶 |
| Gall bladder contents | 1.0 | 74.98 | 1.0 | 1.67 | 🗶 | ✓ | 🗶 |
| Heart | 1.3 | 90.26 | 1.5 | 1.35 | ✓ | ✓ | ✓ |
| Kidney | 1.35 | 95.37 | 1.5 | 1.53 | 🗶 | ✓ | 🗶 |
| Large intestine wall | 1.3 | 84.60 | 1.5 | 1.21 | 🗶 | ✓ | 🗶 |
| Large intestine contents | 1.0 | 58.24 | 1.0 | 0.77 | ✓ | ✓ | ✓ |
| Liver | 1.14 | 61.10 | 1.31 | 0.80 | ✓ | ✓ | ✓ |
| Lung | 1.3 | 32.24 | 1.5 | 0.53 | ✓ | ✓ | ✓ |
| Muscle | 1.39 | 80.94 | 1.68 | 1.30 | ✓ | ✓ | ✓ |
| Oesophagus | 1.3 | 89.37 | 1.5 | 1.46 | 🗶 | ✓ | 🗶 |
| Ovaries | 1.3 | 79.88 | 1.5 | 1.41 | 🗶 | ✓ | 🗶 |
| Pancreas | 1.3 | 81.22 | 1.5 | 1.28 | 🗶 | ✓ | 🗶 |
| Skin | 1.89 | 94.39 | 2.15 | 1.38 | ✓ | ✓ | ✓ |
| Small intestine wall | 1.3 | 90.82 | 1.5 | 2.76 | 🗶 | ✓ | 🗶 |
| Small intestine contents | 1.0 | 58.24 | 1.0 | 0.77 | ✓ | ✓ | ✓ |
| Spinal cord | 1.3 | 48.04 | 1.5 | 0.63 | 🗶 | ✓ | 🗶 |
| Spleen | 1.14 | 75.90 | 1.32 | 1.28 | 🗶 | ✓ | 🗶 |
| Stomach wall | 1.3 | 89.37 | 1.5 | 1.46 | 🗶 | ✓ | 🗶 |
| Stomach contents | 1.0 | 58.24 | 1.0 | 0.77 | ✓ | ✓ | ✓ |
| Thymus | 1.3 | 69.83 | 1.5 | 1.00 | 🗶 | ✓ | 🗶 |
| Thyroid | 1.3 | 81.22 | 1.5 | 1.28 | ✓ | ✓ | ✓ |
| Uterus | 1.3 | 86.17 | 1.5 | 1.56 | 🗶 | ✓ | 🗶 |

**Supporting Information Table S2**. Permittivity and conductivity values used in the neonate models. The permittivity and conductivity ratios are newborn tissue value/adult tissue value at 297 MHz based on data from Peyman et al (ref 15 in paper). Adult tissue values are from Hasgall et al (ref 19 in paper). The right hand column indicates the segmented tissues within each neonate model.

| Simulation | Minimum step (mm) | | | Maximum step (mm) | | | Number of cells |
| --- | --- | --- | --- | --- | --- | --- | --- |
|  | x | y | z | x | y | z | (x 10^6^) |
| Neonate A (adult) | 0.69 | 0.63 | 1.0 | 66.2 | 66.2 | 64.7 | 7.5 |
| Neonate A (neonatal) | 0.69 | 0.69 | 1.0 | 66.2 | 66.2 | 64.7 | 8.1 |
| Neonate B (adult) | 0.69 | 0.69 | 1.0 | 66.2 | 66.2 | 64.7 | 9.2 |
| Neonate B (neonatal) | 0.69 | 0.63 | 1.0 | 66.2 | 66.2 | 64.7 | 9.3 |
| Neonate B (simplified) | 0.69 | 0.63 | 1.0 | 66.2 | 66.2 | 64.7 | 8.8 |
| Duke | 0.69 | 0.69 | 1.0 | 48.5 | 66.2 | 64.7 | 70 |

**Supporting Information Table S3**. Details of mesh settings for EM simulations at 297 MHz

**
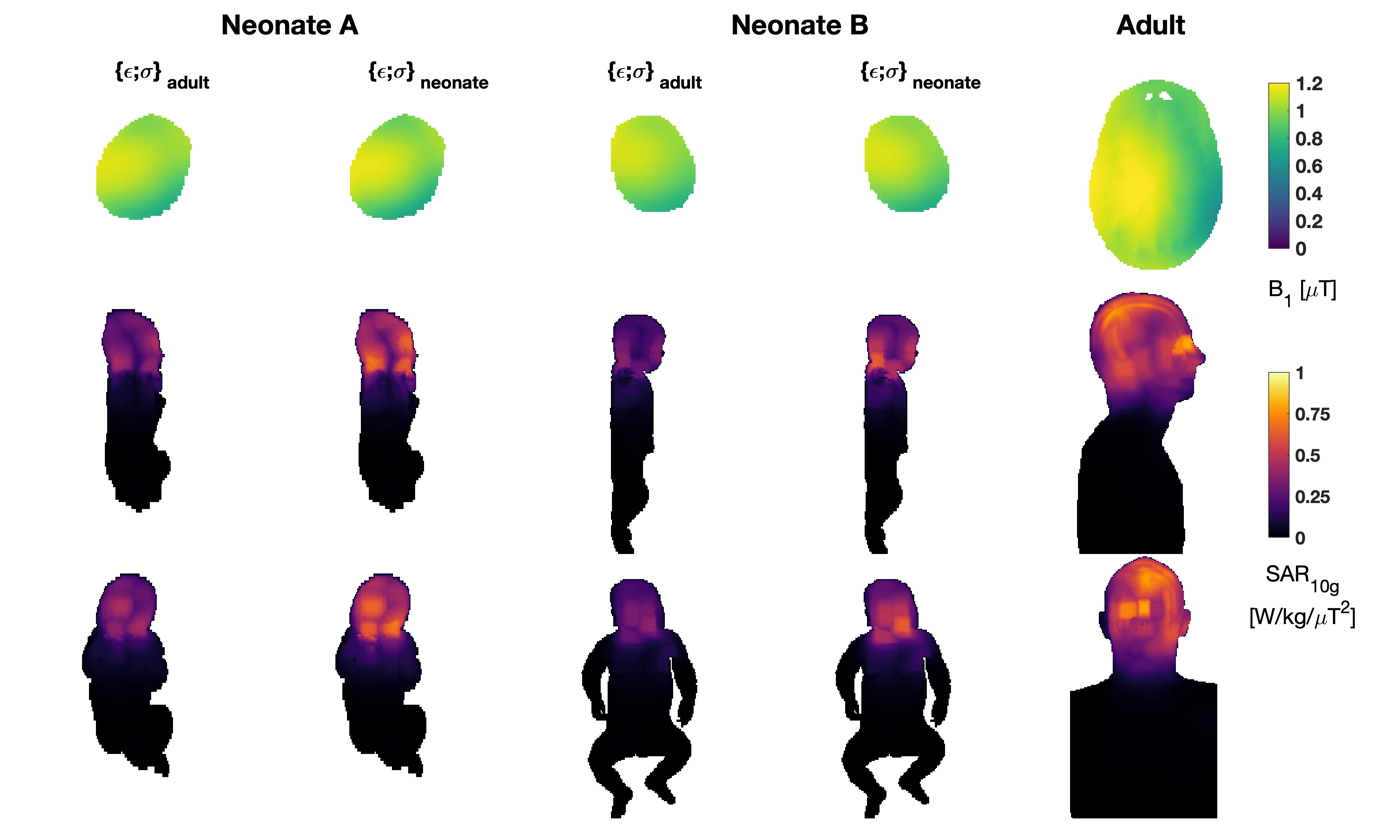
**

**Supporting Information Figure S1**: B_1_^+^ and SAR distributions from the head coil, re-tuned to 127 MHz. The mode identified for these simulations is circularly polarised; the B_1_^+^ field _­_in the adult model has the expected ‘centre brightening’ but is slightly asymmetric left to right. These results are provided to allow comparison with previously published 3T data, but should not be interpreted as representative of a real 3T device.


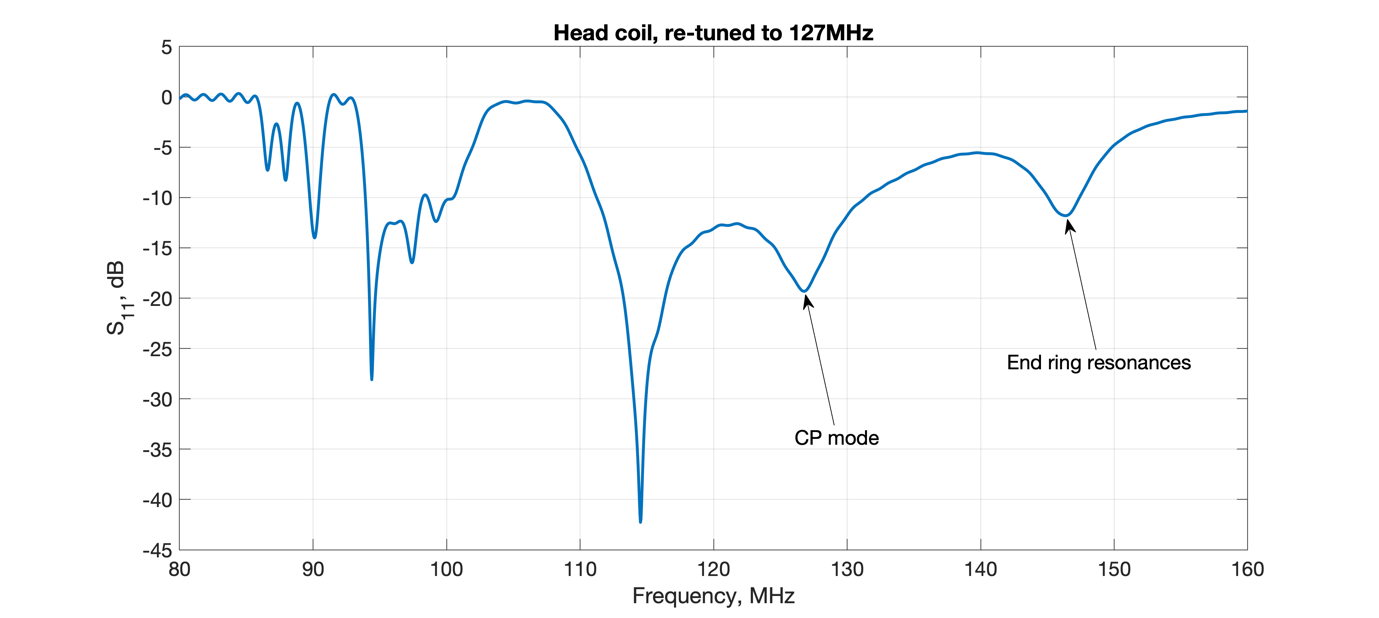


**Supporting Information Figure S2**: S_11_ for retuned birdcage model, from wide-band excitation when loaded with the Duke adult model. The CP mode is identified as the resonance indicated, occurring at 127MHz.

**
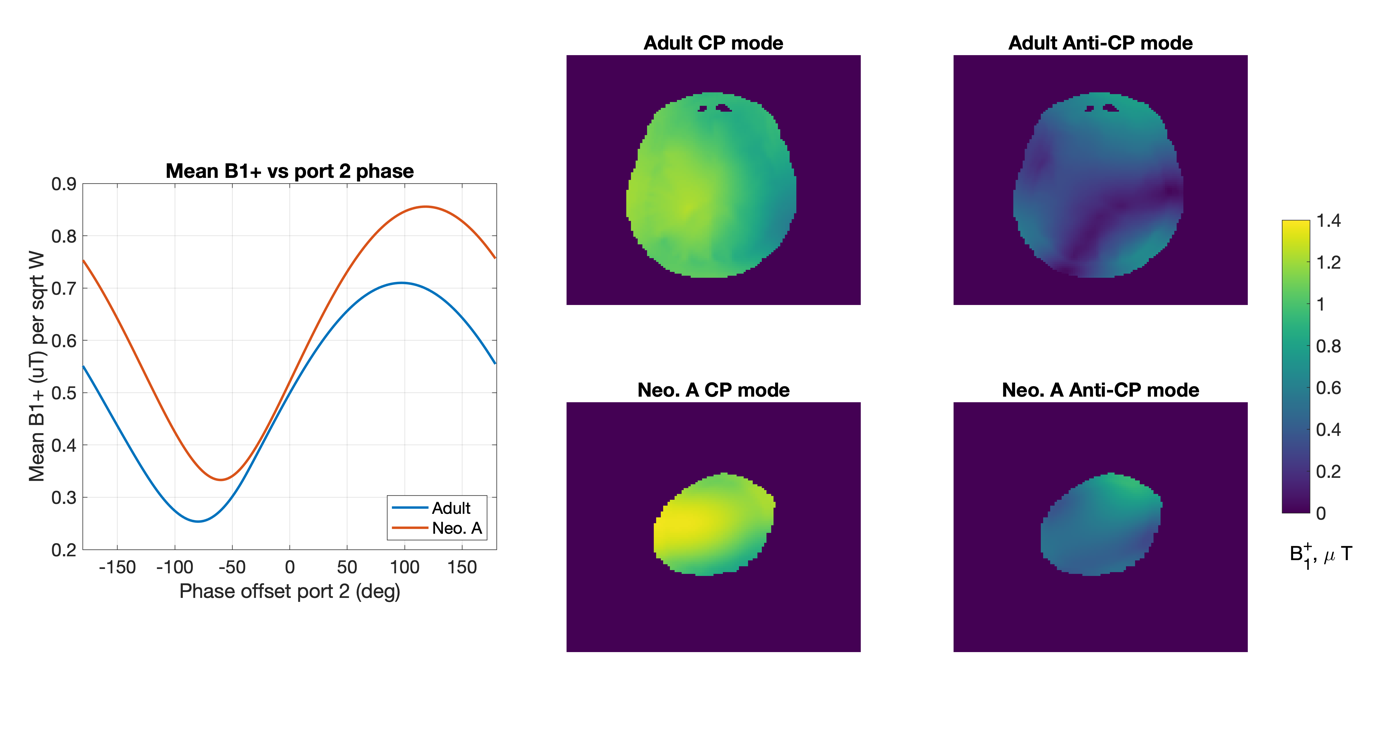
**

**Supporting Information Figure S3:** Investigation of B_1_^+^ distributions from the retuned (127MHz) head coil as a function of phase difference between ports. We expect that a phase difference of +90° would result in a CP mode (high B_1_^+^) while phase difference of -90° would give anti-CP mode (low B_1_^+^). The plots show the mean B_1_^+^ in adult and neonate models as the phase difference is changed, and confirm the expected sinusoidal variation with a minimum at -90° and maximum at +90° for the adult model (for which the coil was tuned). The neonate model sees a small phase shift such that a better CP mode may be achieved with phase shift 120° between ports. To remain consistent with the 7T study in which the coil was set up for the adult and not specifically altered to give better performance in the neonate, a 90° phase shift was used for all presented data.

**
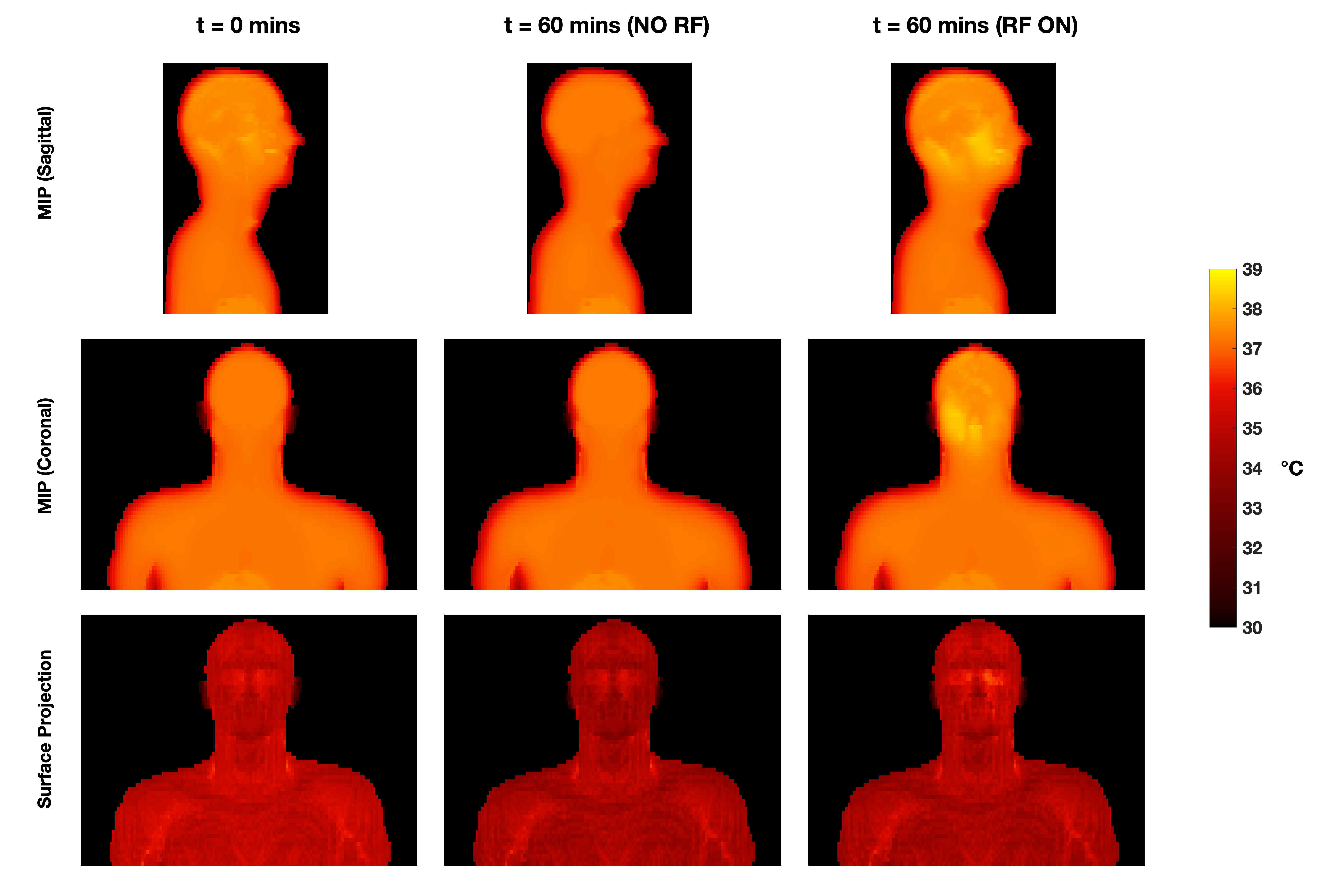
**

**Supporting Information Figure S4**: Thermal simulation results on the adult model (Duke) showing maximum temperature projections (top two rows) and a surface temperature render (bottom row). Results show that after 60 minutes of continuous RF exposure at the SAR limit, the maximum temperature occurs in the head but below the level of the brain. The peak temperature (see Figure 6) is below 39°.
